# Genetic developmental disability diagnosed in adulthood: a case report

**DOI:** 10.1186/s13256-020-02590-8

**Published:** 2021-01-25

**Authors:** Adam Langenfeld, Lynn Schema, Judith K. Eckerle

**Affiliations:** 1 Division of Clinical Behavioral Neuroscience, Department of Pediatrics, University of Minnesota, 717 Delaware Street SE, Minneapolis, MN 55414 USA; 2 Division of Pediatric Genetics and Metabolism, Department of Pediatrics, University of Minnesota, 606 24th Avenue S, Suite 500, Minneapolis, MN 55454 USA; 3 Division of General Pediatrics and Adolescent Health, Department of Pediatrics, University of Minnesota, 717 Delaware Street SE, Minneapolis, MN 55414 USA

**Keywords:** Developmental disability, Intellectual disability, Genetic testing, Chromosome 5, Cri-du-chat syndrome

## Abstract

**Background:**

Developmental disabilities (DD) are an umbrella term for conditions associated with functional impairments in physical, learning, language, or behavior areas. Intellectual disability (ID) is a type of developmental disability that results in delays in cognitive or intellectual functioning, such as reasoning, learning, and problem-solving, and adaptive behaviors including social and practical life skills. DD can be due to a variety of factors, ranging from environmental exposures to genetic mutations, and studies suggest that up to 40% of DDs may be caused by genetic issues.

**Case presentation:**

In this case study, we present an 18-year-old internationally adopted female Chinese American patient with a known history of developmental delay, intellectual disability, strabismus, and a congenital heart defect who had not been tested for genetic causes of her delay prior to presentation. When evaluated with chromosomal microarray, the patient demonstrated a deletion on the short arm of chromosome 5, an area associated with Cri-du-chat syndrome. This chromosomal deletion was a likely explanation for her history of developmental delays, intellectual disability, and congenital heart defect, in addition to her history of institutionalization and the trauma of multiple caregiver transitions in early childhood. The patient was referred for further evaluation by a geneticist and genetic counselor.

**Conclusions:**

This case highlights that the underlying cause of developmental delay is often multifactorial, and underscores the importance of a full medical evaluation, including genetic testing, for children with intellectual disability. Using this approach, healthcare professionals can identify potential diagnoses and provide more targeted resources to families.

## Background

Developmental disabilities (DD) is an umbrella term for conditions associated with functional impairments in physical, learning, language, or behavior areas [1]. Intellectual disability (ID) is a type of developmental disability that results in delays in cognitive or intellectual functioning, such as reasoning, learning, and problem-solving, and adaptive behaviors including social and practical life skills. DD can be due to a variety of factors, ranging from environmental exposures to genetic mutations, and studies suggest that up to 40% of DDs may be caused by genetic issues [2].

Children may demonstrate delays in specific areas, such as speech or fine motor delays, or may have deficits in all areas of growth and development, a condition referred to as global developmental delay. In children who have DDs, identification of the underlying cause of their delays can be challenging. The underlying cause of DDs can be due to a variety of factors, including differences in caregiver interactions, environmental or toxin exposures, and genetic mutations, among others. In addition, DDs often arise from multifactorial causes, making identification of the important contributing factors particularly challenging.

When evaluating children for DDs, practitioners must take a systematic approach to ensure that different contributing factors are considered before making a definitive diagnosis. Current guidelines from the American Academy of Pediatrics (AAP) [3] and American College of Medical Genetics (ACMG) [4] recommend the use of genetic testing, in addition to thorough medical, family, and social histories and a detailed physical exam. Children with a clinical diagnosis of ID should have a comparative genomic hybridization (CGH) microarray to identify copy number variants that can be associated with DD or ID [5, 6]. In addition, guidelines recommend testing for Fragile X syndrome, the most common inherited cause of ID. Thorough past medical, family, and social histories and a detailed physical exam can help identify concerning features that will guide more specific testing.

Although current practice guidelines often help physicians identify underlying causes for DD and ID, 60% of children with DDs do not have an identifiable cause [7]. A child who has previously received a clinical diagnosis of DD or ID may benefit from re-evaluation and additional testing, as the knowledge base surrounding genetic disorders continues to expand.

In this case study, we describe an 18-year-old female patient being seen in the Adoption Medicine Clinic at the University of Minnesota. This patient had a diagnosis of intellectual disability as well as identified developmental delays while living in an orphanage in China prior to adoption, but she had not had previous genetic testing. This case highlights the importance of including genetic testing in the workup of a child with ID to identify underlying genetic causes of these delays, regardless of age or other environmental factors.

## Case presentation

The patient was an 18-year-old Chinese American female being evaluated in the Adoption Medicine Clinic (AMC) at the University of Minnesota. She had been adopted at age 3 years from China. Prior to adoption, she had lived in an orphanage after being relinquished by her biological parents at the age of 5 months. Her previous medical history included a ventricular septal defect (VSD) that was repaired prior to adoption and bilateral strabismus that had been repaired during childhood. She had a resting bilateral hand tremor that was diagnosed as psychogenic by Pediatric Neurology. There was a history of delayed developmental milestones, including gross motor and speech delays. The patient did not start walking until approximately age 24 months. She did not start speaking in phrases or sentences until age 4. Following adoption, the patient’s adoptive mother attempted to obtain resources to help support the patient’s growth and development. She received occupational therapy services from age 3 to 4 years, but this had been discontinued due to lack of insurance coverage. Throughout childhood the family had difficulty obtaining appropriate therapeutic support, and the patient was moved to multiple school districts to identify and obtain resources. She was able to secure financial support from county services at age 16. Despite these challenges, the patient was able to continue to generally progress with her developmental milestones.

The patient had a history of intellectual disability. She had a school individualized education program (IEP) evaluation at age 13 that revealed a full-scale intelligence quotient (IQ) of 70. She was provided school services under the category Developmental Cognitive Disability, mild-moderate. She was evaluated by Pediatric Neuropsychology at age 15 due to ongoing concerns for intellectual disability and developmental delays and was diagnosed with mild intellectual disability and static encephalopathy. She was subsequently evaluated by Pediatric Neurology, who agreed with these diagnoses. The patient also underwent diagnostic testing for autism spectrum disorder (ASD) at age 15, which was not consistent with an ASD diagnosis.

As the patient reached adolescence, she began to have significant difficulty with emotional regulation and mood and was diagnosed with severe depression. She was hospitalized on two separate occasions due to concerns for anxiety, depression, self-injurious behaviors, and suicidal ideation. She was referred for psychotherapy and had been maintained on mood-stabilizing medications, including fluoxetine and escitalopram. She was taking escitalopram at the time of evaluation. The patient previously had dialectical behavioral therapy (DBT) and eye movement desensitization and reprocessing (EMDR) therapy. She had also been treated for an eating disorder at an outpatient program. Throughout adolescence, she continued with social isolation and self-injurious behaviors, including skin picking and cutting, although her mood had improved following initiation of medications and therapy. She had recently started with a mental health coach prior to her evaluation. The patient and adoptive mother had ongoing attachment difficulties. The patient also reported a history of poor sleep related to anxiety and use of electronics prior to bedtime. She was taking melatonin.

At the time of her visit to the AMC, despite the presence of known global delays, diagnosis of mild intellectual disability, and a history of congenital cardiac disease, the patient had not undergone genetic testing. The family had established contact with the patient’s biological family. There was no known consanguinity between parents. She had an older sibling with a hand deformity and a younger sibling without identified medical concerns. Neither sibling was reported to have developmental delays. There was no family history of substance abuse or mental health issues. The patient had an adaptive IEP with occupational therapy support and a plan to transition to adulthood following her senior year of high school. Of note, the patient had spent the majority of ninth grade homebound due to anxiety symptoms and was in a half-day therapy program throughout tenth grade.

On clinical evaluation, the patient displayed appropriate growth and physical development. Weight-for-age was 67th percentile and height-for-age was 37th percentile. Body mass index (BMI) was age-appropriate at 72nd percentile. Vital signs, including blood pressure (91/70) and heart rate (71), were age-appropriate. She did not display any dysmorphic features. She had evidence of self-injurious behaviors, including healed scars on her arms and evidence of skin picking on her scalp. She had a healed surgical incision from her VSD repair. She did not display physical findings consistent with fetal alcohol syndrome. Examination of head and neck was unremarkable. Lungs were clear to auscultation. She demonstrated a quiet systolic heart murmur best heard at her left upper sternal border. Her abdomen was soft and non-tender. Cranial nerves were intact. Deep tendon reflexes were symmetric. She demonstrated appropriate bilateral muscle strength and tone. Laboratory values, including complete blood count (CBC) and vitamin D levels, were within normal limits. She had evidence of stage 2 iron deficiency with low ferritin and elevated iron-binding capacity. She was started on an iron supplement. As part of her clinical workup, the patient was evaluated by a genetic counselor, who recommended obtaining a comparative genomic hybridization (CGH) and single-nucleotide polymorphism (SNP) microarray and testing for Fragile X syndrome, given her history of developmental delays and diagnosis of intellectual disability. She was referred to occupational therapy for management of her sensory sensitivities and fine motor delays.

Chromosomal microarray identified a 6.8 Mb deletion at chromosome location 5p15.31-5p15.33 (arr[GRCh37] arr[GRCh37] 5p15.33p15.31(26142_6858476)x1). This area of chromosome 5 is included in the larger deletion associated with Cri-du-chat syndrome. Although smaller than the typical Cri-du-chat deletion, this patient’s deletion was a likely explanation for her history of developmental delays, intellectual disability, and short stature. It may also have contributed to her congenital heart defect. Interestingly, on further discussion with the adoptive mother and review of medical records, it was found that the patient displayed the characteristic high-pitched cry of Cri-du-chat syndrome during infancy. This patient was referred for further evaluation by a medical geneticist and encouraged to contact her biological family to discuss the genetic testing results and whether any further testing of her siblings was warranted. She was formally seen and assessed by a geneticist and genetic counselor. At that time, additional genetic testing through a karyotype was pursued, which was able to rule out the possibility that this terminal deletion was the result of a translocation. That visit was also critical in determining whether any additional testing or healthcare management was indicated based on the genes included in the deleted region, and to discuss implications for inheritance and future childbearing. She was evaluated by Neurology and underwent brain magnetic resonance imaging (MRI) that revealed no intracranial abnormalities other than a presumed incidental cavernoma in the right thalamus that did not correlate with her symptoms.

## Discussion and conclusions

This case highlights the important role of genetic testing in evaluation of children with developmental disabilities, even when potential causes for delay have been identified. In this case, the patient had been identified as having developmental delays from a young age. She had potential causes for delay in her social history, including unknown social history with her biological parents until age 5 months and institutional neglect related to living in an orphanage until age 3 years. However, despite her diagnosis of intellectual disability and strabismus, as well as an underlying congenital cardiac condition requiring surgical repair prior to adoption, she had not had previous genetic testing. This delay in testing may have been due to healthcare and academic professionals attributing her delays to her history of institutional neglect and multiple caregiver transitions in early childhood rather than an underlying genetic cause. When she underwent genetic testing at the age of 18 years, she was found to have a deletion on chromosome 5. It was only after diagnosis and subsequent investigation that her history of a characteristic cat-like cry during infancy was revealed. This case demonstrates a unique presentation of a genetic cause for developmental delay and supports existing evidence of phenotypic variation related to the size and location of chromosome 5 deletions associated with Cri-du-chat syndrome [8–10].

Developmental disability often results from a combination of factors, and identifying the underlying causes of DDs can be challenging. Even when potential causes of DDs have been identified, it is important to fully investigate for other causes when possible to optimize the resources provided to help improve long-term outcomes for children. Early intervention with a medical diagnosis may provide a child with increased school and county services. Family, school, and professional understanding of a child’s diagnosis and limitations can help normalize behaviors and expectations and potentially limit a child’s feeling of “other” and difference from peers, leading to improved psychosocial and self-esteem outcomes. When evaluating a child for ID, the standard of practice includes obtaining broad genetic testing to evaluate for possible genetic causes, in addition to thorough past medical, family, and social histories and detailed physical exam. This is true even for children who have experienced orphanage or foster care. Using this approach, healthcare professionals can identify potential diagnoses and provide more targeted resources to families.

Cri-du-chat syndrome is a congenital syndrome associated with deletion of part of the short arm of chromosome 5 [10–12]. Deletions can vary in size from extremely small to the entire short arm. Most cases arise from *de novo* mutations, although approximately 12% result from unbalanced segregation of translocations or recombination involving a pericentric inversion in one of the parents. It is one of the most common genetic deletion syndromes, with an incidence of 1 in 20,000 to 1 in 50,000 live births. Cri-du-chat syndrome is characterized in young children by microcephaly, round face, hypertelorism, micrognathia, epicanthal folds, low-set ears, hypotonia, and severe developmental delays [10]. The most characteristic feature is a high-pitched, cat-like cry that is considered diagnostic. However, individuals with a deletion confined to 5p15.3 on chromosome 5 have been found to display the cat-like cry without typical dysmorphic and severe developmental features of the syndrome [8, 9]. In this case, the patient’s chromosomal microarray demonstrated a 6.8 Mb deletion at 5p15.31-5p15.33, consistent with this milder phenotype. Thus, the patient’s history of high-pitched cry, cardiac defect, ophthalmological issues, mild neurocognitive issues, and developmental delays were likely secondary to her chromosomal deletion, in addition to her history of institutionalization and the trauma of multiple transitions and neglect in early childhood.

Developmental disability can be due to a variety of factors. Thorough investigation of the underlying causes of intellectual disability includes a detailed medical and social history, physical exam, and use of genetic testing with chromosomal microarray and testing for Fragile X syndrome, the most common cause of inherited intellectual disability. This case highlights the importance of genetic testing in the evaluation of a patient with developmental disability, as well as the fact that even a child with a known history of causative factors for developmental delay will still benefit from comprehensive assessment including genetic testing. Practitioners should utilize genetic testing for patients with identified developmental and intellectual disabilities, even in adolescent and young adult patients for whom potential underlying causes have previously been identified. Results from genetic testing can help guide further evaluation, including imaging and laboratory studies, and management of associated symptoms. Intervention with a medical diagnosis can lead to increased access to county and social services, improved understanding of behaviors in the home and school setting, and better long-term psychosocial and self-esteem outcomes.

## Data Availability

Not applicable.

## Abbreviations

DD: Developmental disabilities
ID: Intellectual disabilities
AAP: American Academy of Pediatrics
ACMG: American College of Medical Genetics
CGH: Comparative genomic hybridization
AMC: Adoption Medicine Clinic
VSD: Ventricular septal defect
DBT: Dialectical behavioral therapy
EMDR: Eye movement desensitization and reprocessing
IEP: Individualized education program
IQ: Intelligence quotient
ASD: Autism spectrum disorder
SNP: Single-nucleotide polymorphism
